# Eye tracking and attentional bias for depressive internet memes in depression

**DOI:** 10.1007/s00221-020-06001-8

**Published:** 2020-12-17

**Authors:** Umair Akram, Jason G. Ellis, Glhenda Cau, Frayer Hershaw, Ashlieen Rajenthran, Mollie Lowe, Carissa Trommelen, Jennifer Drabble

**Affiliations:** 1 Department of Psychology, Sociology and Politics, Sheffield Hallam University, Collegiate Crescent, Sheffield, S10 2BP UK; 2 Department of Psychology, Faculty of Health and Life Sciences, Northumbria University, Newcastle upon Tyne, UK

**Keywords:** Depression, Depressive memes, Internet memes, Selective attention, Attentional bias

## Abstract

Previous research highlights the potential benefits of engaging with depressive internet memes for those experiencing symptoms of depression. This study aimed to determine whether: compared to non-depressed controls, individuals experiencing depressive symptoms were quicker to orient and maintain overall attention for internet memes depicting depressive content relative to neutral memes. *N* = 21 individuals were grouped based on the severity of reported depression symptoms using the PhQ-9. Specifically, a score of:  ≤ 4 denoted the control group; and  ≥ 15 the depressive symptoms group. Participants viewed a series of meme pairs depicting depressive and neutral memes for periods of 4000 ms. Data for the first fixation onset and duration, total fixation count and total fixation and gaze duration of eye-movements were recorded. A significant group x meme-type interaction indicated that participants with depressive symptoms displayed significantly more fixations on depressive rather than neutral memes. These outcomes provide suggestive evidence for the notion that depressive symptoms are associated with an attentional bias towards socio-emotionally salient stimuli.

## Introduction

The experience of depression is predominately characterized by disordered affect, paired with significant cognitive, emotional and behavioral deficits (American Psychiatric Association 2013). These symptoms are highly prevalent in the general population, affecting up to 27% of individuals according to meta-analytic data (Wang et al. 2017). Cognitive models of depression highlight the role of disorder consistent cognitive biases of information processing (i.e., attention, perception, memory) in the development and maintenance of the disorder (e.g., Beck 1976).

Whilst attentional biases based on reaction time measures are observed in this population, the evidence base remains mixed due to particular methodological differences (Mogg and Bradley 2005). Using the emotional Stroop and dot-probe tasks, studies largely confirm an attentional bias in depression when stimuli are presented for longer (i.e., ≥ 1 s, Donaldson et al. 2007; Gotlib and Cane 1987; Segal et al. 1995) relative to shorter (e.g., 500 ms; Bradley et al. 1995; Donaldson et al. 2007; Mogg et al. 1993) durations. Given the nature of these outcomes, it is theorized that relative to control subjects, depressed individuals are not more vigilant for negative stimuli but retain longer attention towards such information once fixated (Gotlib and Joorman 2010). That said, these differences may not extend to studies using sad stimuli specifically (as opposed to generally negative or threatening stimuli). Nevertheless, reaction time tasks provide an indirect measure of attentional allocation, which are unable to confirm vigilance and disengagement in the context of attentional bias (Marks et al. 2014). As individuals generally orient their eye-gaze towards stimuli that attract attention (Jonides 1981), exploring where individuals direct and fixate their gaze, using eye-tracking, provides a more direct assessment of visual attention (Godijn and Theeuwes 2003). With this approach, visual attention allocation is continuously recorded throughout stimuli presentation, rather than approximating gaze behaviour prior to a response (Marks et al. 2014). In the context of attentional bias, parameters of eye-movements allow for the differentiation of attentional components (i.e., vigilance, maintenance and disengagement; Yiend and Mathews 2005). With that in mind, whilst recent meta-analytic data examining eye-tracking attentional bias studies confirms that depressed individuals exhibit increased attentional maintenance when observing negative stimuli relative to control subjects (Caseras et al. 2007; Suslow et al. 2020; Kellough et al. 2008; Leyman et al. 2011), differences in initial orientation of attention are yet to be established (Armstrong and Olatunji 2012).

As images can convey more affective information, studies using images and scenes, rather than words, are more likely to elicit an attentional bias in depressed individuals (Duque and Vazquez^2015^; Glaser and Glaser 1989). In this context, using internet memes particularly related to the individual experience of depression as this stimulus may allow extrapolation of previous observations of attentional bias to more relatable and ecological stimuli. Many social media platforms host pages devoted to the observation and distribution of internet memes. These are comprised of an image and short caption, which are directly related to the proximal experience of depression (i.e., depressive memes). To that end, we recently observed perceptual differences between subclinical depressed and non-depressed individuals in their interpretation of depressive memes (Akram et al. 2020). Despite the emotional nature of these memes (e.g., death, suicide, isolation), no differences in valance ratings were observed. Crucially however, depressed individuals reported increased perceptual ratings of humor, relatability, shareability and mood-improvement potential of depressive memes when compared with non-depressed controls. Tentatively, these outcomes suggest that depressive memes may serve to promote: a humorous take on negative experiences; and perceived support by connecting with others experiencing related symptoms (Akram et al. 2020).

Using an eye-tracking paradigm, this study examined whether individuals presenting moderate to severe depressive symptoms and non-depressed controls differ in their visual attention allocation towards depressive memes. Specifically, this exploratory study aimed to determine whether: compared to non-depressed controls, individuals experiencing depressive symptoms were quicker to orient and maintain initial and overall attention for internet memes depicting depressive content relative to control memes. In particular, data concerning the first fixation onset and duration, total fixation count and duration, and total gaze duration of eye-movements were recorded. In line with previous work (Akram et al. 2020), we expect individuals with depressive symptoms to display more attention towards depressive, relative to control memes.

## Materials and methods

### Participants

Members of the general population were recruited using posters around Sheffield Hallam University, and social media. The sample consisted of 21 individuals (mean age = 25.45 years, SD = 7.82 years; 85% female), who were grouped on their severity of reported depressive symptoms. Using the PHQ-9, those presenting a score of: ≤ 4 were assigned to the control group (*n* = 12: mean age = 25.16 ± 9.30; 71% female); and ≥ 15 to the depressive symptoms group (*n* = 9: mean age = 22.42 ± 7.44; 74% female). Those currently undergoing psychiatric treatment and/or did not meet the criteria for either group were not eligible to take part. All participants reported normal or corrected-to-normal vision.

### Materials

#### The patient health questionnaire

Symptoms of depression were determined using the patient health questionnaire (PHQ-9: Kronke and Spiltzer 2002). This nine-item scale examines key features of depression as outlined in the DSM-IV-TR/DSM-5 (APA, 2000; 2013). Each item is scored using a four point likert scale (0 = not at all, 1 = several days; 2 = more than half of the days; 3 = nearly all days). A total score is calculated by summating all items. Total scores consequentially range between 0–27, with higher scores representing greater symptom severity. When grouped, 0–4 indicates none-minimal severity, 5–9 mild, 10–14 moderate, 15–19 moderately severe and 20–27 severe depression. Internal consistency (Cronbach’s *α*) in the present study was 0·89.

#### Pictorial stimuli

**Fig. 1:**
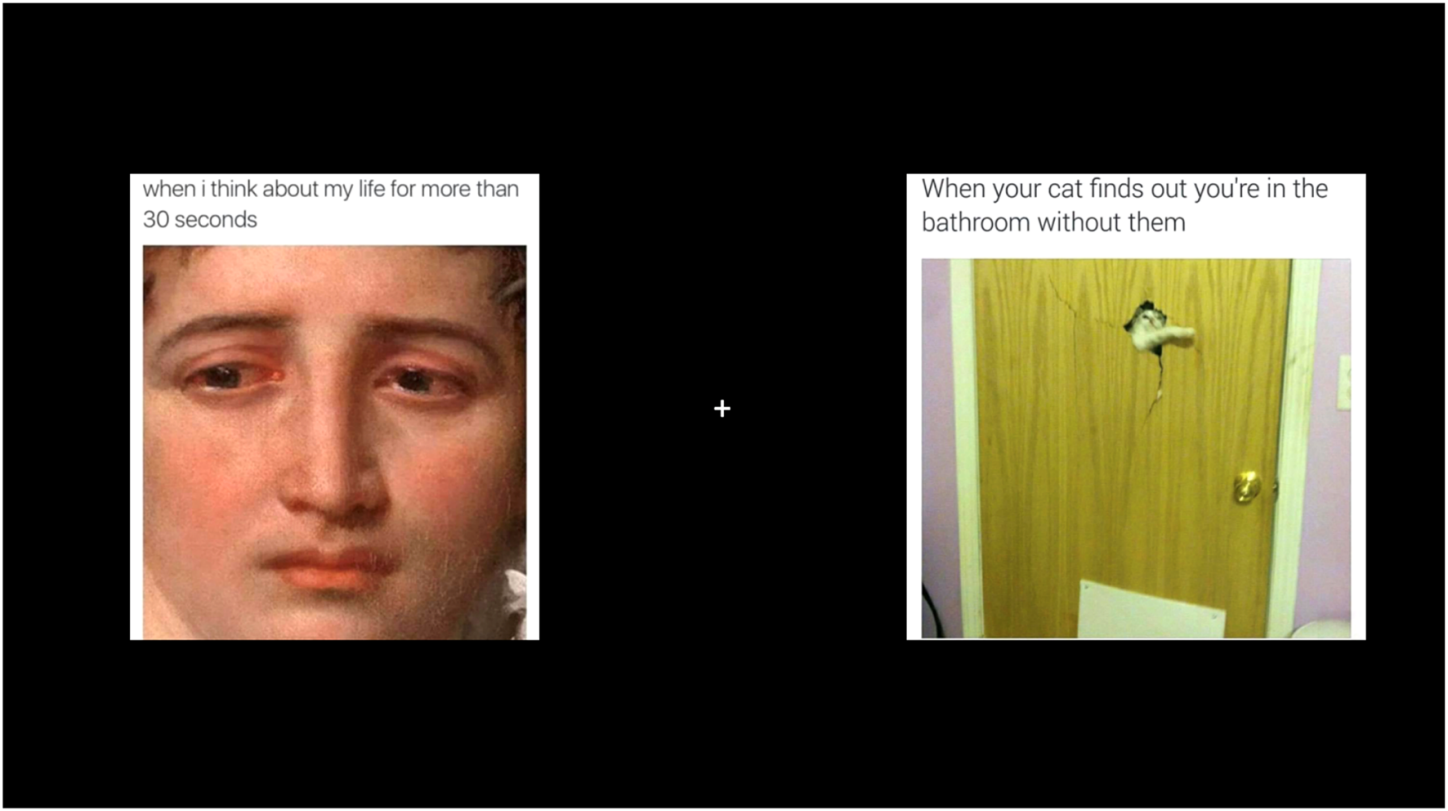
Example trial presenting depressive (left) and control (right) meme-pair used for visual attention task. Of note, the crosshair disappeared on the appearance images and is presented in the current context to highlight the centre point. Images were collected from the public domain and were not modified. They were marked with the Public Domain Mark 1.0 or CC0 1.0 Universal licence. Title and Artist: Unidentified. Image source: snappygoat

We used a previously validated image set comprised of 16 control and 16 depressive memes (see Akram et al. 2020). The depressive memes contained affective content directly related to key features of depression (e.g., suicide, isolation, hopelessness, hypersomnia) indicated by the DSM-5 criteria for major depressive disorder (American Psychiatric Association 2013). In contrast, control memes reflected generally humorous social commentaries that were unrelated to depression (see Fig. 1 for example meme pair). The image size was standardized for presentation (640 × 640px).

### Procedure

The study protocol was approved by the Sheffield Hallam University Research Ethics Committee. The current work was completed in line with the Declaration of Helsinki, and written consent was provided by all participants.

The experiment was conducted in a laboratory room. Gaze behavior was recorded using a Tobii TX300 (Tobii, Stockholm, Sweden) desktop eye tracker with an in-built 21.5 screen used for stimuli presentation. A six-point calibration task was first completed which confirmed < 1° visual angle of eye movement recording. Participants were asked to fixate on a dot appearing center screen. Next, each fixation dot sequentially emerged, and participants were asked to focus on and follow each dot until successful calibration was indicated. Once calibrated, the experimental phase began. During each trial, participants were presented with a pair of meme images (comprising of one control and one depressive meme) for 4000 ms and were asked to observe the memes naturally. At the start of each trial, a fixation cross appeared center screen for consistent starting gaze location across each trial and participant. The ordering of stimuli presentation was counter balanced and randomized. A total of *N* = 32 trials were completed, where each individual meme was displayed twice. Following task completion, participants were debriefed about the nature of the experiment.

### Statistical analyses

#### Interest regions

To assess participant observation of control-depressive meme pairs, two interest regions for each type of meme were predetermined and analyzed for first fixation onset, first fixation duration, total fixation count, total fixation duration, and total gaze duration in milliseconds (ms). Here, fixations are defined significant periods of time where the eyes remain still, holding central foveal vision in place such that the visual system may absorb detailed information from observed stimulus. For each interest region (time measured in milliseconds): first fixation onset was defined as the amount of time elapsed before the first fixation landed within the interest-region; first fixation duration was defined as the time between the start of the first fixation which landed within the interest region until this fixation oriented elsewhere; total fixation count was defined as the elapsed time between the first gaze point and the last gaze point in the sequence of gaze points that makes up a fixation landing within the interest region; total fixation duration was defined as the total summation of the duration of each fixation made within the interest region; and total gaze duration was defined as the time elapsed between the start of the first fixation within the interest region and the end of the last fixation in this region.

Two interest region types were created for each meme (depressive vs. control meme). Specifically, eye movements falling in the inner area of each displayed meme was analyzed in milliseconds. Each variable was extracted using the “Tobii Studio” software (version 3.4.8, Tobii, Stockholm, Sweden). SPSS (version 24, IBM Corp) was used to perform statistical analyses of gaze behavior.

#### Analysis

A series of two (group: control vs. depressive symptoms) × 2 (meme type: depressive vs. control) mixed measures analyses of variance were employed, with first fixation onset, first fixation duration, total fixation duration, and total gaze duration as dependent variables. This was conducted to assess the main effects of group and meme-type. The group × meme-type interactions were also assessed. Tests for pairwise comparisons were also conducted. Significance was considered at the *p* < 0.05 level.

## Results

Mean scores for first fixation onset, first fixation duration, total fixation duration and total gaze duration for each group are presented in Table 1. The internal consistency of each outcome variable is also presented in Table 1. The depression symptom group scored significantly higher (18.44 ± 3.47) on the PHQ-9 than controls (3.83 ± 2.25), confirming group profiles (*t*(19) =  −  11.72, *p* < 0.001). Between groups differences are presented in Table 1, and within group differences in Table 2.

**Table 1:** Scores and between group differences for first fixation onset, first fixation duration, total fixation duration, and total gaze duration (means ± standard deviation) for each group

	Control group	Depression symptoms	*α*	*F*	*P*	Cohen’s *d*
First fixation onset						
Control memes	976 ± 256	1004 ± 257	0.64	0.06	0.804	0.11
Depression memes	959 ± 178	1089 ± 199	0.66	2.50	0.131	0.69
First fixation duration						
Control memes	227 ± 39	197 ± 28	0.84	3.79	0.067	0.88
Depression memes	959 ± 178	1089 ± 199	0.37	2.50	0.131	0.69
Total fixation count						
Control memes	7.97 ± 1.20	6.76 ± 1.88	0.83	3.24	0.088	0.76
Depression memes	6.94 ± 1.34	8.34 ± 1.23	0.90	5.99	0.024^a^	1.09
Total fixation duration						
Control memes	2019 ± 329	1455 ± 434	0.65	11.54	0.003^b^	1.46
Depression memes	1684 ± 359	1927 ± 410	0.95	2.09	0.164	0.63
Total gaze duration						
Control memes	2055 ± 352	1484 ± 426	0.92	11.34	0.003^b^	1.46
Depression memes	1705 ± 355	1969 ± 420	0.92	2.44	0.135	0.68

Note *α* indicates internal consistency of the outcome variables as measured by the Cronbach’s alpha

^a^Sig at *p* < 0.05, ^b^*p* < 0.01

**Table 2:** Within-subjects comparisons of first fixation onset, first fixation duration, total fixation duration, and total gaze duration between each meme-type for the control and depression symptoms group (means ± standard deviation)

	Control Memes	Depression Memes	*t*	*p*	Cohen’s *d*
Control group					
First fixation onset	976 ± 256	959 ± 178	0.24	0.817	0.07
First fixation duration	226 ± 39	959 ± 178	− 13.47	0.001^a^	5.68
Total fixation count	7.97 ± 1.20	6.94 ± 1.34	1.78	0.103	0.81
Total fixation duration	2018 ± 329	1684 ± 359	2.00	0.071	0.77
Total gaze duration	2055 ± 352	1705 ± 355	2.02	0.069	1.00
Depression symptoms group					
First fixation onset	1004 ± 257	1089 ± 199	− 1.35	0.213	0.37
First fixation duration	197 ± 28	1089 ± 199	− 12.66	0.001^a^	6.28
Total fixation count	6.76 ± 1.89	8.34 ± 1.23	− 1.88	0.97	1.00
Total fixation duration	1455 ± 434	1927 ± 410	− 1.97	0.084	1.47
Total gaze duration	1484 ± 426	1969 ± 420	− 1.94	0.088	1.15

^a^Sig at *p* < 0.01

### First fixation onset

The results demonstrated no significant effects of group [*F*(1.19) = 0.86, *p* = 0.365], meme-type [*F*(1.19) = 0.46, *p* = 0.506], or group x meme-type [*F*(1.19) = 1.05, *p* = 0.320] interactions concerning first fixation onset.

### First fixation duration

The results revealed a significant main effect of meme-type [*F*(1.19) = 344.90, *p* = 0.001] on first fixation duration. Pairwise comparisons demonstrated that all participants initially fixated on the depression meme (1089 ± 199 ms) for longer than control memes (197 ± 28 ms). No significant effects of group [*F*(1.19) = 1.58, *p* = 0.224] or group x meme-type [*F *(1.19) = 3.34, *p* = 0.083] interaction was observed.

### Total fixation count

The results demonstrated no significant effects of group [*F*(1.19) = 0.60, *p* = 0.809) or meme-type [*F*(1.19) = 0.31, *p* = 0.587] concerning total fixation count. A significant group x meme-type interaction [*F*(1.19) = 6.98, *p* = 0.016] was demonstrated, indicating that individuals with depressive symptoms demonstrated significantly more fixations on depression memes (8.34 ± 1.23) relative to control memes (6.76 ± 1.87), when compared to individuals in the control group (control memes 7.97 ± 1.20; depressive memes 6.94 ± 1.34).

### Total fixation duration

The results demonstrated no significant effects of group [*F*(1.19) = 3.29, *p* = 0.085] or meme-type [*F*(1.19) = 0.32, *p* = 0.631] concerning total fixation duration. A significant group x meme-type interaction [*F*(1.19) = 8.12, *p* = 0.010] was demonstrated, indicating that individuals with depressive symptoms demonstrated significantly longer total fixation durations for depressive (1927 ± 410 ms) relative to control memes (1455 ± 434 ms), when compared to individuals in the control group (control memes: 2018 ± 329 ms; depressive memes: 1684 ± 359 ms).

### Total gaze duration

The results demonstrated no significant effects of group [*F*(1.19) = 3.35, *p* = 0.083] or meme-type [*F*(1.19) = 0.21, *p* = 0.652] concerning total gaze duration. A significant group x meme-type interaction [*F*(1.19) = 8.05, *p* = 0.011 see Fig. 2) was demonstrated, indicating that individuals with depressive symptoms spent more time observing the depressive (1968 ± 420 ms) relative to control memes (1484 ± 426 ms), when compared to individuals in the control group (control memes: 2055 ± 352 ms; depressive memes: 1705 ± 355 ms).

## Discussion

This study examined whether: compared to non-depressed controls, individuals experiencing depressive symptoms were quicker to orient and maintain initial and overall attention for internet memes depicting depressive content relative to neutral memes. The significant interaction concerning total fixation count evidenced that individuals in the depressive group presented a greater number of fixations on depressive, rather than control memes, when compared with non-depressed controls. In contrast, interactions of total fixation duration and total gaze duration suggest that individuals in the depressive group were less interested in observing the control memes when compared with non-depressed controls. Between groups analyses demonstrated that individuals with depressive symptoms displayed a significantly greater number of fixations on depression, but not control, memes when compared with control subjects. In contrast, whilst no significant within subjects differences were found in either group, the effect size for those in the depression symptoms group was notably large (*d* = 1.00). In relation to total fixation and gaze duration, whilst no between group differences in the observation of depressive memes emerged, individuals with depressive symptoms were significantly less interested in observing the control memes. Whilst no significant within subjects differences were evidenced for either group, effect sizes for those with depressive symptoms were large for measures of total fixation (*d* = 1.47) and gaze duration (*d* = 1.15). Overall, these outcomes cautiously suggest that depression may be associated with alterations in cognitive processing, whereby attentional allocation towards emotionally salient stimuli (i.e., internet memes specifically related to the proximal experience of depression) is altered in this population (Armstrong and Olatunji 2012; Epp et al. 2012). In this case, we provide mixed evidence of an attentional bias for disorder salient internet memes amongst those experiencing symptoms of depression.

Disorder consistent biases of attentional allocation are implicated in the development and maintenance of depression (e.g., Beck, 1976). That said, the potential bias for depressive internet memes may prove to be beneficial for those experiencing symptoms of depression. Indeed, emerging research tentatively highlights perceived benefits associated with online interaction with affective memes (Akram et al. 2020; Kariko and Anasih, 2017). In a survey of 133 college students, 47% of individuals reported engaging with memes as a way of alleviating psychiatric symptoms (Kariko and Anasih, 2017). Here, humor and relatability associated with dark and self-deprecating memes were reported to function as a coping mechanism. Specifically, by allowing individuals to laugh at their problems whilst forming a connection with others in the same situation. More recently, a comparison of depressed individuals and controls evidenced perceptual differences in the interpretation of internet memes illustrating the experience of depression (e.g., death, suicide, isolation, hopelessness, hyper/insomnia: Akram et al. 2020). In particular, the perception of humor, relatability, shareability and mood-improving potential of these memes were significantly higher amongst those presenting with depressive symptoms. Engaging with internet memes related to one’s specific difficulties may potentially come with beneficial consequences. Specifically, by facilitating a humorous take on a negative experience and situation, and the perception of peer-support through affiliation with others experiencing similar symptoms. Although affective memes present a dark and negative humor style, their proximal nature to those experiencing psychiatric symptoms may be considered contextually positive. The experience of psychiatric difficulties for many can be difficult to verbalize. Affective memes, therefore, allow individuals to also visualize the experience and encumbering nature of their symptoms (Akram et al. 2020).

Individuals experiencing depressive symptoms spent less time observing the control memes when compared to individuals in the control group. This disinterest may be explained by a number of factors. Depression appears to negatively influence the cognitive (i.e., impaired accuracy) and affective (i.e., blunted humour ratings) understanding of humour (Falkenberg et al. 2011). In this context, perceived humour related to control memes may have been susceptible to cognitive and affective blunting. However, depressed individuals may differ in their notion of positive and negative humour compared non-depressed people. Indeed, negatively oriented humour may appeal more to this population when considering its relatability and salience to the experience of depression. As depressive internet memes have previously been evidenced to be more humorous and relatable to those experiencing consistent symptoms (c.f., Akram et al. 2020), it is perhaps not surprising that the content of the control memes (i.e., generally humorous social commentaries that were unrelated to depression) may of less interest when compared with individuals in the control group. Certainly, further research is now warranted to clarify the possible role of humour styles and potential group differences in the humour perception of each meme.

Whilst our sample was categorically well split, the total number of participants (*n* = 21) was small. This may have limited our ability to detect interaction effects at the 5% alpha level. However, effect sizes comparing the two groups concerning total gaze duration towards depressive (*d* = 0.68) and neutral memes (*d* = 1.46) were in the large range, suggesting substantial effects. Moreover, many preliminary attentional bias studies using free-viewing eye-tracking paradigms note similar effects with equally small size samples (Stevens, Rist and Gerlach 2011; Eizenman et al. 2003; Felmingham et al. 2011).

In summary, the present outcomes partially support the notion that depression is associated with heightened attentional allocation (i.e., an increased number of fixations) towards depressive internet memes. However, although significant interactions were observed for measures of total fixation and gaze duration, further analysis failed to confirm an attentional bias for depressive memes in the depression symptoms group. Whilst this may be attributed to the sample size, these preliminary findings should now be replicated amongst a larger clinical sample. Finally, as individuals with depression may engage with depressive memes outside of the laboratory setting, a more naturalistic approach should be taken moving forward (i.e., eye movement examination of internet memes embedded into webpages).
